# Retraction Note: Enhancement of the blue photoluminescence intensity for the porous silicon with HfO_2_ filling into microcavities

**DOI:** 10.1038/s41598-020-63852-5

**Published:** 2020-04-23

**Authors:** Ran Jiang, Xianghao Du, Weideng Sun, Zuyin Han, Zhengran Wu

**Affiliations:** School of Physics, Shandong University, Jinan, 250100 China

Retraction of: *Scientific Reports* 10.1038/srep15574, published online 27 October 2015

The authors are retracting this Article because of duplication of findings and figures from previously published studies [1,2] and issues with assembly of figures.

Specifically, the production of porous-Si filled with HfO2, and the finding that HfO2-filled porous-Si enhances blue light emission have been reported by the authors in a previous publication [1], which was not cited in the Article. The inset in Fig. 2 is duplicated from Fig. 1b in [1], the spectra in Fig. 2 are previously published as Fig. 2a in [1], the data in Fig. 5 are published as Fig. 3 in [1], the information presented in Fig. 6 is published as Fig. 4 in [1], and the data in Fig. 7 have been presented in Fig. 2b of [1]. Additionally, Fig. 3b is duplicated from Fig. 1c in [2].

All Authors agree with this retraction.
